# Quality of Life Assessment in Patients with Multiple Sclerosis Receiving Interferon Beta-1a: A Comparative Longitudinal Study of Avonex and Its Biosimilar CinnoVex

**DOI:** 10.5402/2012/786526

**Published:** 2012-08-09

**Authors:** R. Abolfazli, A. Hosseini, Kh. Gholami, M. R. Javadi, H. Torkamandi, S. Emami

**Affiliations:** ^1^Neurology Department, Amiralam Hospital, Tehran University of Medical Sciences, Tehran 1145765111, Iran; ^2^Islamic Azad University, Tehran, Iran; ^3^Faculty of Pharmacy, Tehran University of Medical Sciences, Tehran, Iran

## Abstract

*Background*. Multiple sclerosis (MS) is an autoimmune inflammatory disease of central nervous system (CNS). MS affects quality of Life (QOL) due to physical disability and other associated problems. Disease-modifying agents like interferon beta (IFNB) have been widely utilized in this patient population; however, their frequency, route of administration, side effects, high cost, and also the question of whether they are truly beneficial for longer-term outcomes and QOL need to be further investigated. *Objectives*. To assess QOL in patients with multiple sclerosis receiving interferon beta-1a (Avonex or CinnoVex) and in order to compare QOL in groups receiving Avonex and CinnoVex, respectively, also, to evaluate whether the more cost-effective biosimilar form of IFNB (CinnoVex) has the same effect on QOL and can be substituted for Avonex. *Methods*. We conducted a 30-month, nonrandomized longitudinal study and recruited a total of 92 patients diagnosed with relapsing-remitting MS. The patients were distributed in Avonex and CinnoVex groups with 46 patients in each group. Quality of life was assessed by means of MSQOL-54 questionnaire, four times a year, at baseline and at months 4, 8, and 12 of the study. *Results*. Mean age ± SD was 30.5 ± 8.9 and 32.3 ± 9.0 years in Avonex and CinnoVex groups, respectively, and *P* value of gender was different (*P* value : 0.036). The physical health composite scores were 61.8 and 59.8 (*P* values 0.677 and 0.884) for Avonex and CinnoVex groups, in that order. The results of the study revealed no significant difference between the two groups with regard to physical health, health perception, energy, and role limitations due to physical problems, pain, sexual and social function, and physical health distress scores. Further, interferon therapy did not significantly impact patients' QOL after a year of treatment with either Avonex or CinnoVex. *Conclusions*. According to the present study, treatment with IFNB (Avonex or CinnoVex) did not affect QOL during a year of therapy. Further studies with longer follow-up periods are required to assess the value of interferons on long-term outcomes and patient's QOL.

## 1. Introduction

Multiple sclerosis (MS) is an autoimmune inflammatory disease of central nervous system (CNS) characterized by demyelination and axonal damage [1]. MS is the leading cause of neurological disability in younger adults and is defined as periods of acute attacks (relapses), progressive deterioration, or both [2, 3]. Between 250,000 and 300,000 people suffer from MS in the United States, and the annual healthcare costs are estimated to be $10 billion [4, 5]. Quality of Life (QOL) is affected by MS due to physical disability and other associated problems such as cognitive dysfunction, visual impairment, and pain [6]. Quality of life is a multidimensional concept encompassing physical, social, psychological, and emotional functions [7]. Health-related quality of life (HRQOL) is defined as those aspects of life function that are affected by patients' health status and its quantification assists clinicians in assessing disease progression as well as effects of drug therapy [8, 9].

Several tools and questionnaires have been utilized to assess HRQOL in MS. The short form 36 (SF-36) [10] is a tool to measure a generic HRQOL; however, more disease-specific scales should supplement SF-36. Other tools such as Expanded Disability Status Scale (EDSS) [11], Hamburg Quality of Life Questionnaire in multiple sclerosis (HAQUAMS, German) [12] or multiple sclerosis quality of life questionnaire (MSQOL54) [13] are reliable instruments widely utilized for QOL assessment in MS.

Although it is documented that disease-modifying agents such as interferons are beneficial for short-term outcomes in MS, they might negatively affect disability outcomes and the overall QOL due to their route and frequency of administration as well as several side effects [14]. In a few previous studies, interferon beta (IFNB) was shown to have a negative affect on QOL, mostly by increasing fatigue and depression [15, 16]. Adherence to IFNB therapy is also a great challenge for clinicians with rates of drug discontinuation ranging from 9 to 21 percent in clinical trials [2].

CinnoVex, a generic biosimilar form of IFNB-1a, was recently approved in Iran. In the previous studies with CinnoVex, it was documented to slow disease progression and disability and in some cases showed improvement in the course of the disease [17].

The objectives of the present study were to: (1) assess QOL in patients with MS receiving interferon beta-1a (Avonex and CinnoVex) and (2) evaluate and compare QOL in groups receiving Avonex and CinnoVex.

## 2. Materials and Methods

### 2.1. Setting

The study was conducted from April 2009 to September 2011 (for the duration of 30 months) in Amiralam hospital, a 226-bed teaching affiliate of Tehran University of Medical Sciences (TUMS).

The hospital consisted of internal, surgical, coronary care, and intensive care units and is one of the pioneers and most equipped centers for treatment of ear, nose, and throat (ENT) diseases in the country.

### 2.2. Patients and Design

A nonrandomized, longitudinal study was conducted on 92 patients diagnosed with relapsing-remitting multiple sclerosis (RR-MS). Selected patients did not receive any type of interferon in the last 6 months preceding recruitment, and the EDSS scores were between 0–4. Exclusion criteria were current or past history of psychiatric disorders, two clinical relapses (attacks) within a 6-month period, pregnancy, and history of serious drug-related side effect such as elevated liver enzymes.

Written informed consent was obtained from patients meeting inclusion/exclusion criteria and who were willing to participate in the study. Patients' demographic data were obtained at two stages; primary demographics including age, sex, marital status, age at disease onset, age disease diagnosed, and family history of MS were obtained at baseline whereas secondary demographics such as the one-year change in financial situation, employment status, level of education, education regarding their disease, and change in family status were collected after completion of the study (at month 12). Patients were then equally distributed in either Avonex or CinnoVex groups according to the neurologist's consultation (at inclusion time, selected patients in either group did not differ in EDSS, demographics, or QOL). QOL assessment was conducted four times in a 12-month period, at baseline (stage 1) and at months 4 (stage 2), 8 (stage 3), and 12 (stage 4) following recruitment and by means of MSQOL54 [13]. Clinical disability was measured using the EDSS [11]. Before filling the questionnaire at each stage, patients were examined by the neurologist, and the EDSS scores were obtained. The examinations included mental, cranial, motor system, sensory, cerebellar, gait, and stance exams. The MSQOL54 questionnaire consisted of 54 items from generic short-form 36 (SF-36) [18] and 18 additional MS-specific questions with each question scoring between 0–100. Physical and mental health composite scores were calculated using the weight sum of selected items. Patients' scores in either mental or physical health sections were determined as follows: (1) at the first stage, the scores obtained from each question in physical or mental health sections were added up, and the mean was calculated (final scaled score), (2) the obtained mean from the first stage was multiplied by its specific coefficient or weight (subtotal), and (3) the final mental or physical scores were obtained from their specific scores from stage 2 and physical and mental health composites were retrieved.

Additionally, in order to assess between-group variations in Avonex and CinnoVex groups, statistical tests were performed at all 4 stages of the study. Independent sample *t* test (to assess EDSS scores), Chi-square test (to assess cerebellar, sensory, and gait stance scores), and Mann-Whitney *U* test for assessing motor system, cranial, and mental scores. Further, within-group assessments were obtained by using ANOVA repeated measure test for both Avonex and CinnoVex groups. All tests were performed using SPSS version 19 (SPSS Inc., Chicago, IL).

## 3. Results

**Figure 1:**
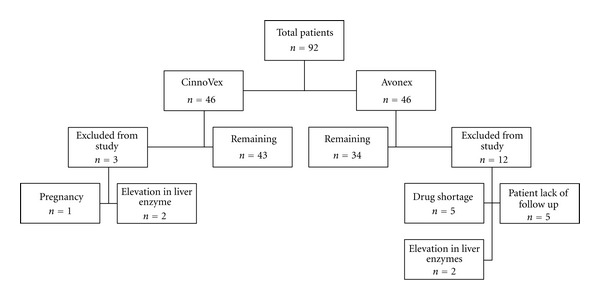
Population distribution of the studied patients.

We recruited 92 patients in the study and 14 patients were excluded from the study in different stages. The final analysis was conducted on the 77 remaining patients with 34 and 43 patients in Avonex and CinnoVex groups, respectively (Figure 1).

The mean ± SD age of Avonex group was 30.5 ± 8.9, and in CinnoVex group was 32.3 ± 9.0. Most of patients were between 20–30 years of age. Patients' primary and secondary demographic data are summarized in Table 1. The mean physical health composites were 61.8 and 59.8 at baseline and 63.1 and 59.8, for stages 2–4, (*P* values 0.677 and 0.884) in Avonex and CinnoVex groups, accordingly. The mean mental health composites for Avonex and CinnoVex groups were 57.4 and 53.6 at baseline and 55.1 and 55.2, for stages 2–4, (*P* values 0.197 and 0.845). Moreover, assessment of individual factors affecting physical or mental health did not reveal a significant difference between the two groups (Tables 2 and 3).

The results from the four stages of physical examination and EDSS scores revealed no significant difference between Avonex and CinnoVex groups (*P* values 0.206 and 0.702). Among primary and secondary demographic factors included in regression analysis for Avonex group, only the change in financial situation had a statistically significant effect on final physical and mental health scores (*P*-value 0.050) whereas in CinnoVex group, a change in patient's settlement had such an effect (*P*-value 0.026). Factors affecting mental and physical health scores in Avonex and CinnoVex groups are compared against each other in Table 4.

## 4. Discussion

Compared to other chronic diseases, multiple sclerosis, especially in its progressive form, has the most notable effect on HRQOL according to studies conducted worldwide [19–22]. Physical dysfunction and social limitations have the most unfavorable effect on MS patients' QOL, and half of all patients are unable to conduct their household and employment responsibilities within 10 years of disease onset [23]. The present study was the first study to evaluate the effect of interferon beta-1a therapy on QOL in MS patients in Iran. According to the results, interferon beta therapy did not change QOL after one year of treatment. Moreover, we found no significant difference in QOL between groups of patients receiving Avonex or CinnoVex.

The overall well being of patients with chronic illnesses such as MS is not a simple measurement of disability but rather a concept encompassing both physical and psychosocial aspects of QOL [8]. Even though measures of disability, such as EDSS, have been correlated with HRQOL, this correlation is weak, varying from 2–29% [23]. Additionally, disability scales, such as EDSS or Multiple Sclerosis Functional Composite (MSFC) [24], have limitations since they rely on assessment by the neurologist rather than patients' self-assessment or adjustment to the illness, which have the strongest correlation with HRQOL [8]. Further, in HRQOL assessment, response shift can negatively affect results of longitudinal studies by changing patient's perception of the disease over time, leading to underestimation of treatment effectiveness [25]. Although MSQOL-54 is widely accepted as a MS-specific instrument for QOL assessment, it has limitations such as notable floor and ceiling effects (limiting its use in wheelchair-bound patients), lack of sensitivity, and the time to complete MSQOL-54 [26]. Other HRQOL instruments such as MSQLI [27] and MusiQOL [28] have advantages and limitations as well. MusiQOL is particularly a reliable instrument with the advantage of being based on patients' views and perceptions as well as being shorter; however, lack of data regarding its use in disease-modifying drug studies has limited its utility [26].

Immunological treatment has variable effects on HRQOL and some studies have shown no significant improvement in patients' overall QOL [29]. Rice et al. [29], concluded that IFNB improves patients' physical ability, and Simone et al. [15] reported that IFNB has a negative impact on QOL after 2 years of treatment, influencing mainly mental QOL. Vermersch, et al. [16] also conducted a research on RR-MS patients undergoing treatment with Avonex and stated that QOL is correlated with disability in MS, and IFNB treatment has no negative effect on patients' QOL. According to our study, no significant difference was observed between patients' QOL at baseline and after a year of therapy with IFNB. The true estimation of patients' QOL depends on assessment of immunological treatment as well as MS-specific QOL instruments. Although emerging immunological therapies may have improved efficacy for the patient, they might negatively affect QOL due to their more serious side effects or their possible lack of benefit on HRQOL [15]. By overcoming limitations of instruments and development of a more simplified QOL instrument with higher validity and subjectivity, the true effect of immunological treatment can be more accurately and reliably assessed.

According to the present study, a slight improvement in QOL was observed at stage 2 (month 4) of the study compared to the baseline; however, it declined to the baseline at the end of stage 4 (month 12). The improvement in QOL at the end of stage 2 could be due to patients' initial positive perception towards treatment with IFNB, and the misconception that interferons cure their disease. The observed decline in QOL at stages 3 and 4 (back to the baseline) probably indicates that patients' expectations of treatment were not met. To overcome this issue, patients should be educated regarding true effects of immunological treatment on MS course and QOL.

CinnoVex was shown in the previous studies to slow disease progression and control relapses in MS patients with similar side effect profile to other IFNBs [17], although it is a more cost-effective form of IFNB-1a in comparison with Avonex. According to the present study, we observed no significant difference between groups of patients receiving Avonex or CinnoVex with regard to overall QOL (either physical or mental health composite scores). Therefore, the more cost-effective IFNB (CinnoVex) could be substituted for Avonex in RR-MS patients; however, further study is required with CinnoVex since it is a recently approved drug with limited postmarketing data including side effect and safety profile.

In addition, the short follow-up period (as compared to previous longer-term longitudinal studies) [7, 15] represents a limitation of the present study. Further, for a more accurate assessment of effects of IFNB therapy on QOL, larger numbers of treated patients are required, however, we could not do so due to limitations of the study.

In the future studies, patients' psychological well-being should always be taken into account and larger patient population should be recruited. Moreover, the impact of interferons' side effects on QOL should be assessed in order to draw a more reliable conclusion of effects of interferons on patients' QOL.

## Figures and Tables

**Table 1:** Demographic data of the studied patients.

Parameter		Avonex (*n* = 34)	CinnoVex (*n* = 43)	*P* value
Age, year (mean ± SD)		30.5 ± 8.9	32.3 ± 9.0	
	<20, *n* (%)	4 (11.8%)	0	
	20–30, *n* (%)	15 (44.1%)	20 (46.5%)	0.203
	30–40, *n* (%)	11 (32.3%)	16 (37.2%)	
	>40, *n* (%)	4 (11.8%)	7 (16.3%)	
Sex, female, *n* (%)		31 (91.1%)	31 (72.0%)	0.036
Marital status, single, *n* (%)		18 (52.9%)	18 (41.8%)	0.333
Age at disease onset, year (mean ± SD)		26.1 ± 9.0	29.4 ± 8.7	0.165
Family history of MS, *n* (%)		2 (5.9%)	5 (11.6%)	0.384

**Table 2:** Factors affecting physical health scores of the studied patients.

Factor		Avonex			CinnoVex	
	Baseline	Stages 2–4	*P* value	Baseline	Stages 2–4	*P* value
Physical health	12.8	13.1	0.587	12.2	12.4	0.475
Health perception	9.9	10.1	0.559	9.9	9.4	0.127
Energy/fatigue	6.2	5.9	0.185	5.7	5.8	0.541
Role limitations due to physical problems	7.6	7.4	0.914	6.3	6.3	0.958
Pain	7.6	7.8	0.640	7.5	7.4	0.185
Sexual function	1.7	1.9	0.411	2.6	2.8	0.585
Social function	8.2	8.5	0.516	8.3	8.3	0.877
Physical health distress	7.4	7.3	0.757	6.9	7.0	0.685
Physical health composite	61.8	63.1	0.677	59.8	59.8	0.884

**Table 3:** Factors affecting mental health scores of the studied patients.

Factor		Avonex			CinnoVex	
	Baseline	Stages 2–4	*P* value	Baseline	Stages 2–4	*P* value
Emotional health distress	9.4	9.3	0.757	8.8	8.9	0.685
Overall quality of life (QOL)	6.2	8.7	0.536	5.9	6.1	0.733
Emotional well-being	16.1	15.9	0.713	15.3	15.9	0.205
Role limitations due to emotional problems	13.8	13.0	0.844	12.0	11.9	0.896
Cognitive function	11.2	10.5	0.016	10.6	10.1	0.050
Mental health composite	57.4	55.1	0.197	53.6	55.2	0.845

**Table 4:** Comparing factors affecting physical and mental health in Avonex and CinnoVex groups.

Time-varying covariate		Avonex			CinnoVex		*P*	
	Baseline	Change^∗^	*P*	Baseline	Change	*P*	Baseline	Change
EDSS	1.9	0.1	0.489	1.5	0.2	0.401	0.104	0.231
MSQOL-54 measures								
Physical health composite score	61.8	1.2	0.677	59.8	0.0	0.884	0.608	0.377
Mental health composite score	57.4	−2.2	0.197	53.6	1.6	0.845	0.392	0.841
Physical function	12.8	0.2	0.587	12.2	0.2	0.475	0.465	0.324
Health perception	9.9	0.2	0.559	9.9	−0.5	0.127	0.932	0.251
Energy	6.2	−0.25	0.185	5.74	0.1	0.541	0.316	0.828
Role limitation—physical	7.6	−0.2	0.914	6.3	−0.0	0.958	0.223	0.201
Bodily pain	7.6	0.1	0.640	7.5	−0.17	0.185	0.850	0.356
Sexual function	1.7	0.27	0.411	2.6	0.2	0.585	0.172	0.189
Social function	8.2	0.2	0.516	8.3	−0.0	0.877	0.904	0.756
Health distress—physical	7.4	−0.1	0.757	6.9	0.0	0.685	0.434	0.587
Health distress—mental	9.4	−0.1	0.757	9.3	0.1	0.685	0.434	0.587
Overall quality of life	6.2	2.4	0.536	5.9	0.1	0.733	0.505	0.491
Cognitive function	11.2	−0.6	0.016	10.6	−0.5	0.050	0.494	0.548
Emotional well-being	16.1	−0.2	0.713	15.3	0.6	0.205	0.516	0.995
Role limitation—emotional	13.8	−0.8	0.844	12.0	−0.1	0.896	0.442	0.544
Satisfaction with sexual function	19.1	5.6	0.221	30.2	1.5	0.698	0.169	0.368
Change in health	48.5	1.9	0.669	50.5	4.2	0.275	0.760	0.418

^∗^ Change after a year.
